# Cross-lagged associations among family-supportive supervisory behaviors, leader–member exchange, and work engagement: a test of gain spirals of resources

**DOI:** 10.1186/s40359-026-04349-0

**Published:** 2026-03-17

**Authors:** Hai Jiang, Jiyuan Mei, Jinhua Gan, Jing Luo

**Affiliations:** 1 Wuhan Municipal Institute of Public Administration, Wuhan, 430000 China; 2 School of Business, Central South University, Changsha, 410083 China; 3 Department of Psychology, College of Education and Sports Sciences, Yangtze University, Jingzhou, Hubei 434023 China

**Keywords:** Family-supportive supervisory behaviors, Leader-member exchange, Work engagement, Cross-lagged, The conservation of resources

## Abstract

Based on the Conservation of Resources Theory, this study examines the reciprocal relationships between family-supportive supervisory behaviors (FSSB), leader–member exchange (LMX), and work engagement. Its primary aim is to clarify not only the forward pathway from FSSB to LMX and then to work engagement, but also the reverse effects from work engagement back to LMX and FSSB—a bidirectional perspective seldom explored in prior research. Using a two-wave longitudinal design with a six-month interval, data were collected from 286 full-time employees across six supermarkets in China. Structural equation modeling was employed to test the proposed model. The results reveal positive reciprocal relationships between FSSB and LMX, as well as between LMX and work engagement. These findings suggest that FSSB fosters high-quality LMX, which subsequently enhances work engagement, while engaged employees also tend to perceive and elicit more supportive supervision over time. The study contributes to the literature by empirically validating dynamic interplays among FSSB, LMX, and work engagement, offering practical insights for developing supportive leadership and enhancing employee engagement in organizational settings.

**Supplementary Information:**

The online version contains supplementary material available at 10.1186/s40359-026-04349-0.

## Introduction

It has been widely acknowledged that the resource investment of supervisors will, in turn, increase the resource investment of employees [1]. For instance, supportive supervisory behavior predicted employee positive work behavior and improved work performance [2–4]. Those researchers focused on resource sources and effects but neglected how resources flow among each other [5], and few studies have discussed how the resource engagement of individual employees invariably prompts supervisors to provide more work resources [6, 7].

To address this gap, the current study adopts the Conservation of Resources (COR) theory to explain the dynamic, reciprocal resource flow between supervisors and employees. COR theory posits that individuals strive to obtain, retain, and protect valued resources, and that those with greater resources are more capable of gaining further resources, leading to “gain spirals” [8]. Within this framework, Family-Supportive Supervisory Behaviors (FSSB) constitute a critical work resource provided by supervisors. The prioritization of FSSB by organizations stemmed from its ability to meet employees’ diverse work-family demands [9]. This support helped employees fulfill their obligations in both domains, thereby prompting them to devote more resources to work [10, 11]. However, the process is likely not unidirectional. COR theory suggests the potential for reciprocal gains, yet the dynamic, interpersonal process whereby supervisory investment initiates a gain spiral that eventually benefits the supervisor remains underexplored [12, 13]. Existing research has also found a gain spiral between job resources and job engagement [14].

Thus, we made several significant contributions to the literature on COR theory and processes of resource investment between supervisors and employees.

First, it initiates an inquiry into a specific starting point for resource gain spirals between individuals. While research on gain spirals spans over a decade, consensus on their starting point is lacking [8]. Prior studies often relied on overly general constructs [6, 7]. We argue that FSSB is a theoretically and practically pivotal starting point because it is a core supervisory behavior for assisting employees with work-family demands [9, 15], and (b) represents an alterable behavior with substantial potential returns on organizational investment [9, 16]. This alterability aligns with the notion of tightrope-walking leadership, where supervisors must dynamically balance competing demands such as flexibility (e.g., providing family support) and efficiency [17]. This perspective suggests that the provision of FSSB is not automatic but a contingent leadership resource, responsive to both employee needs and organizational context.

Second, we extend COR theory to model gain spirals through reciprocal interpersonal dynamics [12], specifically via Leader-Member Exchange (LMX). Although research indicates FSSB can increase work engagement indirectly through LMX [10], the potential reverse pathway—whereby employee work engagement enhances LMX and subsequently encourages more FSSB—remains unexamined. Previous gain spiral research has predominantly focused on intra-individual processes [7, 18–20]. Only scarce research has investigated inter-individual cycles [21, 22], and none have applied this lens to the supervisor-employee dyad to explain how resource investment can become mutually reinforcing.

**Fig. 1:**
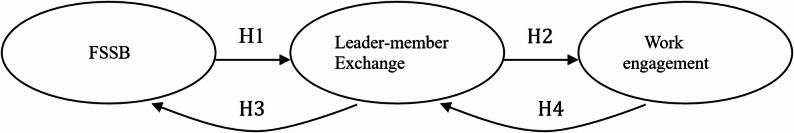
The research model

The current research aimed to use COR theory to explain the dynamic flow between family-supportive supervisor behavior and employee work engagement. As a work resource provided by supervisors, FSSB can prompt the increase of supervisor–employee relationship resources (LMX) and subsequently improve individual work engagement (see Fig. 1). This is a resource flowing process initiated by supervisors that then forms resource caravans. Engagement of employee work resources will further enhance supervisor–employee relationship resources and finally influence supervisors, which leads to supervisors’ investing more resources. It is also a resource recycling. We used cross-lagged panel data from six supermarkets in China to examine the relationship.

### Resource caravans: from FSSB to work engagement

#### FSSB and LMX relationship

COR theory focuses on the notion of resources defined as “entities that either are centrally valued in their own right or act as a means to obtain centrally valued ends” [1]. People are motivated to protect, replenish, and invest resources, which are linked to other resources [23]. This identifies a general tendency for enrichment of resources among those who possess a solid resource reservoir; increasing resources tend to form resource caravans [1].

As a work resource, FSSB means supervisors show their support for the families of their employees [24]. As a means to help employees fulfill their work and family obligations and improve their work–family relationships [25]. Much extant empirical research has confirmed that family-supportive supervisor behaviors (FSSB) not only effectively reduce work-family conflict and promote work-family balance [26–28], but also exert broad positive influences on employee attitudes, behaviors, and health [10, 11, 29–31]. This conclusion is further corroborated by a recent meta-analysis based on the Ability-Motivation-Opportunity (AMO) framework, which indicates that FSSB exhibits significant associations with core work, family, and health outcomes. Notably, FSSB demonstrates unique incremental effects and discriminant validity beyond coworker support, flexible work arrangements, and family-supportive organizational perceptions for critical outcomes such as task performance, burnout, organizational commitment, and job satisfaction [32]. In addition, investigations into work–family practice identified that FSSB could be altered [16, 33]. When FSSB improved, employees developed more positive work attitudes and behaviors.

Supervisory behavior plays a key role in forming high-quality LMX [34–36]. The direct supervisor was a representative of an organization. He/she helped employees satisfy their demands by providing work resources from the organization. Supervisor behavior was thus extremely critical to employee work attitude and behavior shaping [29]. A more critical factor was that supervisor resources that cater to employee demands directly influence the motivation of employees to build high-quality relationships with their supervisor [34].

Family-supportive supervisors helped employees manage their work–family relationship through satisfying social and emotional demands required by employees (such as respect, care, and support), so as to effectively release pressure caused by work–family conflict. They also helped employees better fulfill their work and family obligations [37]. Supportive behavior resulted in more positive employee evaluations of their supervisors [38] and made them more willing to build emotional connections with their supervisors and recognize their relationship with supervisors [39].

These positive impacts were representative characteristics of high-quality LMX, in which supervisors and employees maintain a high level of mutual trust and respect [34]. Research demonstrates that FSSB strengthens the social exchange relationship with supervisors, thereby motivating employees to engage in voice behavior; this mediating effect of LMX is more pronounced when the family role of the employee’s spouse is less salient [40]. Critically, this process is fundamentally perceptual. Employees’ interpretation of supervisory actions forms the basis of their attitudinal and behavioral responses. Research on workplace dynamics confirms that perceptions of the work environment (e.g., of support versus abuse) are pivotal drivers of subsequent attitudes and behaviors, underscoring the importance of examining the perceptual pathways through which FSSB operates [41]. Accordingly, we hypothesize the following:

Hypothesis 1: FSSB has a positive cross-lagged effect on LMX.

#### LMX relationship and work engagement

When employees perceive a high-quality exchange relationship between themselves and their supervisors, they will commit more resources (like job involvement) to benefit their supervisors. Employee work engagement has been defined as a positive, fulfilling, and work-related state of mind that is characterized by vigor, dedication, and absorption [42]. In high-quality exchange relationships, employees regard themselves as members of their supervisors’ coteries. To sustain such a high-quality relationship resource, employees devote themselves to work, that is, the high-level of work engagement to maintain LMX [43]. Previous research has suggested that high-quality LMX could lead to positive organizational behavior [10, 44]. Researchers reckoned that employees who maintain high-quality relationships are engaging in organizational citizenship behaviors with both supervisors and their organizations [10]. Meta-analysis also proved that LMX could significantly predict positive organizational behaviors [45]. Macey and Schneider took organizational citizenship behavior as extra-role behavior, also an expression of work engagement [46]. High-quality LMX would improve employee work motivation, which was formed when recognizing their supervisors [44, 47]. Previous research used social exchange theory and psychological contract theory to discuss the relationship between LMX and work engagement. Social exchange theory holds the view that high-level LMX was, under interdependence, successive interpersonal interaction between supervisors and employees, which formed a sense of accountability in heart of employees [38]. Through work engagement, employees rewarded their supervisors through displaying such accountability. Therefore, work engagement was the form of returning or exchanging [46].

The research showed that LMX could positively predict work engagement and LMX was also a critical part of social exchange relationship in an organization in a sample of 235 Chinese employees reckoned LMX was a work engagement antecedent [48, 49]. In their opinion, the key factor in how employees work was leader behavior. Their meta-analysis indicated the average correlation coefficient between LMX and work engagement was 0.31. Kahn [50] deemed that employees who trust their supervisors devote more to work because they perceive a sense of psychological security. Hypothesis 2 is:

Hypothesis 2: LMX has a positive cross-lagged effect on work engagement.

### Gain resource spirals: from work engagement to FSSB

COR theory holds the view that the process of gaining resources is dynamic rather than stationary and bi-directional rather than mono-directional. Resources continually develop and become “resource convertible” with mutual benefits among different resources. Initial resource gain could prompt further resource so as to shape a reciprocal “gain spiral” [51]. Despite resource gain spiral playing critical roles in the dynamic process described in COR theory, there is little research on this topic [7, 51, 52].

Work engagement is a process of individual resource engagement, and current resource engagement can bring back more resources to gain [23]. For example, Hakanen found that work engagement could improve the acquisition of work–family and work resources [53]. Rodríguez Muñoz found that employee work engagement improved resource gain in family fields, increasing individual happiness [54]. According to the COR theory, LMX is a kind of interpersonal resource, meaning that individual resource engagement could be exchanged for the gain of leader–member resources [1].

Organizations need dedicated employees. As representatives of their organizations, supervisors ask their subordinates to devote themselves more to work. Those employees who devote more to work can potentially achieve higher work performance evaluations and more recognition and identification, so as to perceive a high-level LMX. Work engagement could improve related individual perception of work and result in positive employee behavior [55, 56]. Moreover, LMX is employee interpersonal perception during working hours; positive emotion brought by work engagement can improve interpersonal perception, leading to high-quality LMX. Bagger analyzed the outcome variables of work engagement [10]. They reckoned that contextual performance, as an aspect of work performance, was a critical outcome variable of work engagement. Moreover, contextual performance included the interpersonal interaction between supervisors and subordinates, and interpersonal facilitation was a dimension of contextual performance [57]. The research of Christian demonstrated that work engagement could significantly positively predict contextual performance [48]. We hypothesize the following:

Hypothesis 3: Work engagement has a positive cross-lagged effect on LMX.

Finally, we also assume an effect from LMX to FSSB. Wayne’s research proved that societal support exists in a mutually beneficial relationship with LMX [58]. However, Eisenberger did not verify Wayne et al.’s finding [59]. Their research indicated that societal support predicted LMX, but vice versa was not true. Considering this discrepancy, they explained that support offered by direct supervisors, not ordinary social support, could improve LMX.

High-quality LMX could result in employee perception of more supervisory support [60, 61]. Although Bagger and Li’s research [10] did not take into consideration how LMX improved FSSB further, they mentioned that there may be a kind of mutual beneficial relationship and positive cycle between LMX and FSSB. Straub’s research proposed that an appropriate quality of LMX was an antecedent of FSSB [62]. He conceived that in high-quality exchanges, employees could fulfill the role their supervisors expected of them and, subsequently, the supervisors would assign more challenging tasks and offer more job autonomy. Thereafter, employees perceive family support from their supervisors.

The research conducted by Matthews and Toumbeva demonstrated a mutually beneficial relationship between LMX and FSSB in a short-term (six-week) interval [63]. They reckoned that high-level exchange had existed before supervisors offered family support to employees, which triggered the emergence of FSSB.

According to the mutual beneficial relationship of social exchange theory, when an employee perceives that they have a high-quality relationship with their supervisor, they are more likely to engage in behaviors that benefit the supervisor (i.e., increased work engagement). Supervisors invest more FSSB because they consider the behavior as feedback in high-level exchange. In the view of COR theory, the mutual beneficial relationship between employee work and supervisor provision of interpersonal resources is the process of mutual gain. The gain of interpersonal resources is the intrinsic motivation for supervisors to provide work resources, such as FSSB. Therefore, our fourth hypothesis reads as follows:

Hypothesis 4: LMX has a positive cross-lagged effect on FSSB.

## Materials and methods

### Research procedure and sample

Our study adopted a two-wave longitudinal design with six months interval. Informed consent was obtained from all participants, and the study was approved by the Ethics Committee of the Faculty of Psychology at Yangtze University. We recruited full-time employees from six giant Chinese outlets located in eastern China to complete the offline paper-and-pencil questionnaire survey. By first getting formal permission and support from each supermarket’s top managers, we gained access to employees. This approach minimizes non-response bias and ensures that the survey reaches as many employees across different departments as possible. Participants were asked to report three focal variables: FSSB, LMX, and work engagement level at both time points. In addition, following previous studies [64], we collected participants’ demographic information, including age, gender, tenure, and marital status as control variables. At Time 1, we distributed 641 questionnaires and received 572 responses. After excluding responses with abnormal values or incomplete data on more than two-thirds of key variables, we ended up with 503 valid responses. At Time 2, we invited all former 503 valid participants to complete the follow-up survey and received 340 responses. After conducting the same cleaning procedure at Time 1, a final 286 valid samples were collected. Among these 286 samples, the average age of participants was 34.05 years (SD = 7.3). The sample consisted of 219 women (76.6%), 232 married individuals (81.1%), and 139 participants who had more than 3 years of tenure (49.3%). In addition, we conducted a series of Mann-Whitney U tests and t tests to determine if systematic differences existed across two samples: (a) final sample (*N* = 286); (b) initial sample at T1 (*N* = 503). Results showed that there did not exist significant differences between the excluded sample and the final sample on gender (Z = -1.54, *p* = .12), age (t = -1.93, *p* = .06), tenure (Z = -1.72, *p* = .09), marriage state (Z = -1.76, *p* = .08), FSSB (t = − 0.62, *p* = .54), LMX (t = 0.67, *p* = .51), and work engagement (t = − 0.08, *p* = .94). Results found no significant differences between panel participants and dropouts in demographics or study variables, suggesting no serious selection issues arose from attrition.

### Research instruments

#### Measures

Given that we conducted our survey in mainland China, we employed a rigorous translation and back-translation procedure to adapt the primary scales to the Chinese language. First, the third and fourth authors initially translated the overall English questionnaire into Chinese. Then, we invited two bilingual Ph.D. students who passed the English standard test to translate it back into English. Finally, the four actors compared and discussed any discrepancies to create the final Chinese scale.

Family supportive supervisor behavior (FSSB). This construct was evaluated using Hammer et al.’s 14-item measure [28], which encompasses four subscales: emotional support (α = 0.77 at T1, α = 0.83 at T2), instrumental support (α = 0.79 at T1, α = 0.73 at T2), role modeling (α = 0.86 at T1, α = 0.86 at T2), and creative work-family management (α = 0.86 at T1, α = 0.83 at T2). Participants rated their supervisors’ behaviors on a five-point Likert scale, from “strongly disagree” to “strongly agree.” The global FSSB construct yields a α = 0.91 at T1 and α = 0.92 at T2.

Leader-member exchange (LMX). This construct was assessed with Graen and Uhl-Bien’s [65] seven-item measure (e.g., “How well does your manager understand your job problems and needs?”). The Cronbach’s alphas were 0.85 at T1 and 0.84 at T2. Responses were provided on a seven-point Likert scale (1 = strongly disagree to 7 = strongly agree).

Work engagement. This construct was measured using Schaufeli et al.’s 17-item scale [43], which comprises three subscales: vigor (α = 0.82 at T1, α = 0.83 at T2), dedication (α = 0.92 at T1, α = 0.91 at T2), and absorption (α = 0.87 at T1, α = 0.89 at T2). Participants responded to statements like “I feel vigor when I work” on a seven-point Likert scale, indicating their agreement from “strongly disagree” to “strongly agree.” The global work engagement construct yields a α = 0.94 at T1 and α = 0.94 at T2.

#### Statistical analyses

Referring to previous research [7, 53, 66–68], we utilized structural equation modeling techniques by using the AMOS 26.0 software to test the hypothesized cross-lagged model. As discussed above, we simplified two multi-dimensional constructs, FSSB and work engagement, into four and three indicators, respectively, in the tested models. However, considering that leader-member exchange was represented by one dimension, we kept its seven items as indicators.

In order to test the mediating role of leader-member exchange and obtain the potential spiral effect, we carried out an examination process suggested by Cole and Maxwell (2003) and Taris and Kompier (2006) [66, 68]. Specifically, the current study entailed two analysis parts: first, we tested the relationship between the predictor FSSB and the mediator LMX; second, we examined the causal relationship between the mediator LMX and the outcome variable work engagement, as depicted in Fig. 1. Furthermore, we examined causal and reversed causal effects simultaneously by employing a full panel design and compared several alternative models in each part. Initially, we tested a stability model (Mstabil), which assumes that each latent variable at Time 1 (T1) predicts the same factor at Time 2 (T2) without any cross-lagged associations. We then conducted comparative analyses among Mstabil, a causality model (Mcausal), a reversed causation model (Mreversed), and a reciprocal model (Mreciproc). Take the relationship between FSSB and LMX as an example to illustrate the details. (1) We first established a model that only includes FSSB at T1 predicting FSSB at T2, and LMX at T1 predicting LMX at T2, and named this model M1stabil. (2) Building on this, we created a M1causal model, which added a cross-lagged path from FSSB at T1 to LMX at T2. (3) For directly observing the reversed relationship between focal variables, we only included a reversed cross-lagged path from LMX at T1 to FSSB at T2 based on the M1stabil model to test the reversed causal relationship, naming it M1reversed. (4) Finally, we combined the three models above to construct an overall reciprocal model (named M1reciproc), which suggests a reciprocal effect that FSSB at T1 could influence LMX at T2, and LMX at T1 predicts FSSB at T2. Similarly, we conducted the same cross-lagged analysis procedure when verifying the relationship between LMX and work engagement and named the four models M2stabil, M2causal, M2reversed, and M2reciproc. In addition, according to prior studies’ suggestion [7, 53], we correlated the error term of each indicator of the same variable across time points (e.g., four indicators of FSSB at T1 and T2).

To interpret the magnitude of the estimated cross-lagged effects, we refer to the recently established empirical benchmarks for cross-lagged panel models (CLPM), where effect sizes of 0.03, 0.07, and 0.12 represent small, medium, and large effects, respectively [69]. These guidelines provide a standardized frame of reference for evaluating the practical significance of the prospective relationships in our model.

## Results

### Descriptive statistics

Table 1presents correlations between the study variables. FSSB, felt LMX, and work engagement showed a relatively high rank-order stability over time. FSSB correlated positively with LMX at each wave and across waves: the correlations ranged from 0.63 to 0.75, p < .001. LMX correlated positively with work engagement within and between waves, 0.54 < r < .56, p < .001.

**Table 1:** Means, standard deviations, and correlations between the study

Time 1	M	SD	1	2	3	4	5	6	
1. FSSB	3.80	0.63	1						
2. LMX	4.78	0.89	0.75^***^	1					
3. Work engagement	4.82	0.98	0.49^***^	0.56^***^	1				
***Time 2***									
4. FSSB	3.70	0.59	0.68^***^	0.63^***^	0.44^***^	1			
5. LMX	4.75	0.79	0.63^***^	0.66^***^	0.51^***^	0.70^***^	1		
6. Work engagement	4.73	0.93	0.50^***^	0.54^***^	0.73^***^	0.52^***^	0.62^***^	1	

* Abbreviations: n*  286, *FSSB *family-supportive supervisory behaviors, *LMX *leader–member exchange, ^***^p < .001

### Common method bias

Before the confirmatory factor analyses (CFAs) of the entire model, we additionally validated the factor structure of the FSSB and work engagement construct, as the two constructs contained multiple composites. Specifically, this study conducted two separate CFAs at T1 and T2. Each CFA specified a seven-factor model comprising four first-order factors for FSSB and three first-order factors for WE, as indicated by the original scales. The model fit indices for both time points demonstrated acceptable model fit: T1 (*χ²* = 880.645, *df* = 413, *p* < .001; CFI = 0.92, TLI = 0.91, RMSEA = 0.06) and T2 (*χ²* = 902.253, *df* = 413, *p* < .001; CFI = 0.92, TLI = 0.90, RMSEA = 0.06). These results support the validity of the first-order composites.

The results of the CFAs to test the measurement models are presented in the upper part of Table 2. The T1 hypothesized measurement model including three latent factors (i.e., FSSB, LMX, and work engagement) yielded a good fit to the data: χ2 = 164.293, df = 74, p < .001; GFI = 0.92, CFI = 0.96, TLI = 0.95, and RMSEA = 0.07. Furthermore, this hypothesized measurement model fit the data better than the alternative one-factor model: χ2 = 503.705, df = 77, p < .001; GFI = 0.78, CFI = 0.80, TLI = 0.76; ∆χ2 = 330.412, ∆df = 3, p < .001. None of the alternative models provided a good fit. Similar results were found when testing the T2 measurement models. Furthermore, we conducted an additional CFA to examine the fit of the comprehensive cross-lagged model including FSSB, LMX, and work engagement. The results showed that the measurement model demonstrated an acceptable fit. These results confirm that the comprehensive model remain a good fit to the data: χ2 = 544.272, df = 321, p < .001; GFI = 0.88, CFI = 0.96, TLI = 0.95, and RMSEA = 0.05. In addition, we adopted a full collinearity VIF test method to further identify potential common method bias [70]. Results showed that full collinearity VIFs of all focal variables meets the requirement of lower than a 3.3 threshold [70]. Specifically, full collinearity VIFs of supportive supervisor (FSSB) at T1 and T2 are 2.308 and 2.406 respectively, VIFs of leader-member exchange (LMX) at T1 and T2 are 2.746 and 2.655 respectively, and VIFs of work engagement at T1 and T2 are 2.932 and 2.593 respectively. Combined with the measurement model examination results, we considered that the proposed theoretical model has no significant common method bias.

**Table 2:** ༎Fit statistics for the measurement models

Models	χ2	df	GFI	CFI	TLI	RMSEA	Comparison	Δχ2	Δdf			*p*
Time1												
MM1	164.293	74	0.92	0.96	0.95	0.07	MM2 − MM1	339.412		3	< 0.001	
MM2	503.705	77	0.78	0.80	0.76	0.14						
ULMF1	114.646	60	0.95	0.97	0.96	0.06						
***Time2***												
MM1	197.291	74	0.91	0.95	0.93	0.08	MM2 − MM1	396.24		3	< 0.001	
MM2	593.537	77	0.74	0.77	0.73	0.15						
ULMF2	127.685	60	0.94	0.97	0.96	0.06						

MM1 means the three latent factors model (FSSB, leader-member exchange, and work engagement). MM2 means the single latent factor model (FSSB + leader-member exchange + work engagement). ULMF1 means the model that added an unmeasured latent factor based on three-factor model at Time 1. ULMF2 means the model that added an unmeasured latent factor based on three-factor model at Time 2

### Factorial invariance

We inspected configural invariance, metric invariance, scalar invariance, and error variance invariance of our key constructs separately and the entire model across time. Results are shown in Table 3. Results revealed that the differences of each construct between the configural invariance model, the metric invariance model, the scalar invariance model, and the error variance invariance model were less than the cutoffs (i.e., ∆CFI ≤ 0.01, ∆RMSEA ≤ 0.015). However, for the entire model, the error variance invariance model did not meet the threshold. Following the suggestions of prior research [71, 72], achieving configural, metric, and scalar invariance is generally sufficient to support meaningful comparisons of latent constructs across time. Thus, we concluded that all the constructs and the entire model remain robust across Time 1 and Time 2.

**Table 3:** Results for measurement invariance across time (*n* = 286)

Measurement invariance	χ^2^	df	*p*	CFI	RMSEA	Model comparison	∆CFI	∆RMSEA
FSSB								
Configural invariance	210.202	142	< 0.001	0.98	0.04	-		
Metric invariance	225.4778	152	< 0.001	0.98	0.04	Ma	0.00	0.00
Scalar invariance	241.985	162	< 0.001	0.97	0.04	Mb	0.01	0.00
Error variance invariance	270.468	176	< 0.001	0.97	0.04	Mc	0.00	0.00
LMX								
Configural invariance	70.281	28	< 0.001	0.96	0.07	-		
Metric invariance	78.497	34	< 0.001	0.96	0.07	Ma	0.00	0.00
Scalar invariance	90.274	40	< 0.001	0.95	0.07	Mb	0.01	0.00
Error variance invariance	106.075	47	< 0.001	0.94	0.07	Mc	0.01	0.00
WE								
Configural invariance	557.090	232	< 0.001	0.93	0.07	-		
Metric invariance	581.102	246	< 0.001	0.93	0.07	Ma	0.00	0.00
Scalar invariance	611.066	260	< 0.001	0.92	0.07	Mb	0.01	0.00
Error variance invariance	620.691	277	< 0.001	0.92	0.07	Mc	0.00	0.00
The entire model								
Configural invariance	2105.772	1274	< 0.001	0.92	0.05	-		
Metric invariance	2146.947	1304	< 0.001	0.92	0.05	Ma	0.00	0.00
Scalar invariance	2202.776	1334	< 0.001	0.92	0.05	Mb	0.00	0.00
Error variance invariance	3323.605	1393	< 0.001	0.82	0.07	Mc	0.10	0.02

*Abbreviations*: *χ2 *chi-square test statistic, *CFI *comparative fit index, *RMSEA  *root-mean-square error approximate, *Ma *The row of model difference shows differences between configural invariance model and metric invariance models, *Mb * The row of model difference shows differences between metric invariance model and scalar invariance models, *Mc * The row of model difference shows differences between scalar invariance model and error variance invariance models

### Test of the hypotheses

**Fig. 2:**
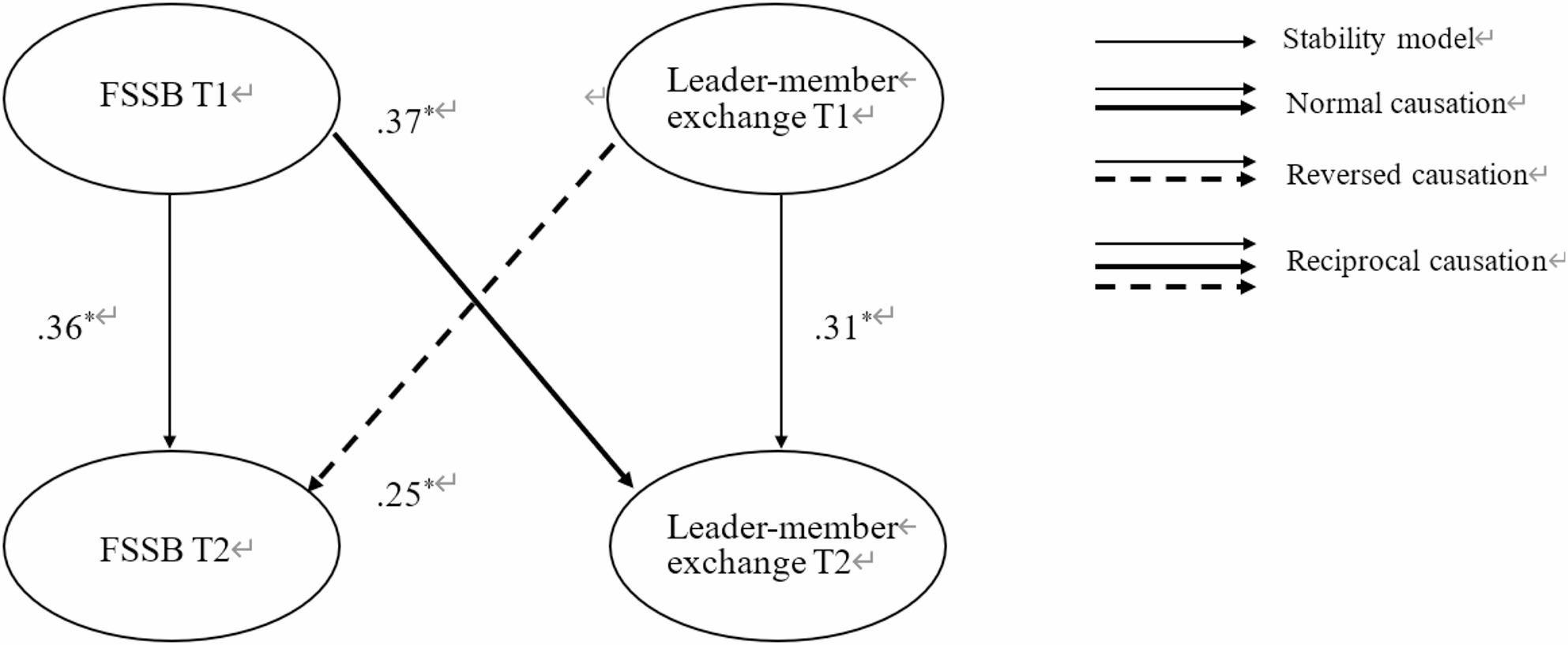
The final model of the statistically significant cross-lagged relationships between FSSB and LMX]

The research model integrated three latent variables, each measured at two points in time with their corresponding indicators. To evaluate our hypotheses, we conducted cross-lagged analyses within a two-step process: first, examining the relationship between Family Supportive Supervisor Behavior (FSSB) and Leader-Member Exchange (LMX), and second, investigating the link between LMX and work engagement [7, 53]. Table 4reveals that the reciprocal model (M1reciproc), which postulates mutual cross-lagged effects between FSSB and LMX, provided a superior fit to the data compared to the stability model (M1stabil; ∆χ^2^ = 14.261, ∆*df* = 2, *p* < .001), the causality model (M1causal; ∆χ^2^ = 5.575, ∆*df* = 1, *p* < .05), and the reversed causation model (M1reversed; ∆χ^2^ = 4.393, ∆*df* = 1, *p* < .05). A further path analysis shown that after controlling the latent variables’ temporal autoregressive effects, employees’ gender, age, tenure and marital status, FSSB at T1 significantly positively predicted LMX at T2 (β = 0.37, SE = 0.17, *p* < .05), and conversely, LMX at T1 significantly positively predicted FSSB at T2 (standardized β = 0.25, SE = 0.10, *p* < .05, see Fig. 2). Consequently, FSSB and LMX were found to exert reciprocal influences over the six-month duration of our study.

**Table 4:** Fit statistics for the study models (*n* = 286)

Model description		χ^2^	df	IFI	TLI	CFI	RMSEA	AIC	Model comparisons	∆χ^2^	∆df	∆RMSEA	∆AIC	*p*-value
**Model Cross-lagged relationships between FSSB and LMX**														
M1 stabil	Stability model	452.410	270	0.95	0.93	0.95	0.05	666.410						
M1 causal	Causality model (M1 stabil + FSSB → LMX)	443.724	269	0.95	0.93	0.95	0.05	659.724	M1 stabil – M1 causal	8.686	1	0.00	6.686	<0.01
M1 reversed	Reversed causation model (M1 stabil + LMX → FSSB)	442.542	269	0.95	0.94	0.95	0.05	658.542	M1 stabil – M1 reversed	9.868	1	0.00	7.868	<0.01
M1 reciproc	*Reciprocal model* *(M1 causal +M1 reversed)*	*438.149*	*268*	*0.95*	*0.94*	*0.95*	*0.05*	656.149	M1 stabil – M1 reciproc	14.261	2	0.00	40.261	<0.001
									M1 causal – M1 reciproc	5.575	1	0.00	3.575	<0.05
									M1 reversed – M1 reciproc	4.393	1	0.00	2.393	<0.05
**Model Cross-lagged relationships between LMX and work engagement**														
M2 stabil	Stability model	376.173	224	0.96	0.94	0.96	0.05	576.173						
M1 causal	Causality model (M2 stabil + LMX → WE)	362.026	223	0.96	0.94	0.96	0.05	564.026	M2 stabil – M2 causal	14.147	1	0.00	12.147	<0.001
M2 reversed	Reversed causation model (M2 stabil + WE → LMX)	364.290	223	0.96	0.94	0.96	0.05	556.290	M2 stabil − M2reversed	11.883	1	0.00	19.883	<0.001
M2 reciproc	*Reciprocal model* *(M2 causal +M2 reversed)*	*354.657*	*222*	*0.96*	*0.95*	*0.96*	*0.05*	*558.657*	M2 stabil − M2reciproc	21.516	2	0.00	17.519	<0.001
									M2 causal – M2 reciproc	7.369	1	0.00	5.369	<0.01
									M2 reversed – M2 reciproc	9.633	1	0.00	2.367	<0.01

Cross-lagged relationships between leader–member exchange (LMX) and work engagement (WE); family-supportive supervisory behaviors (FSSB).

**Fig. 3:**
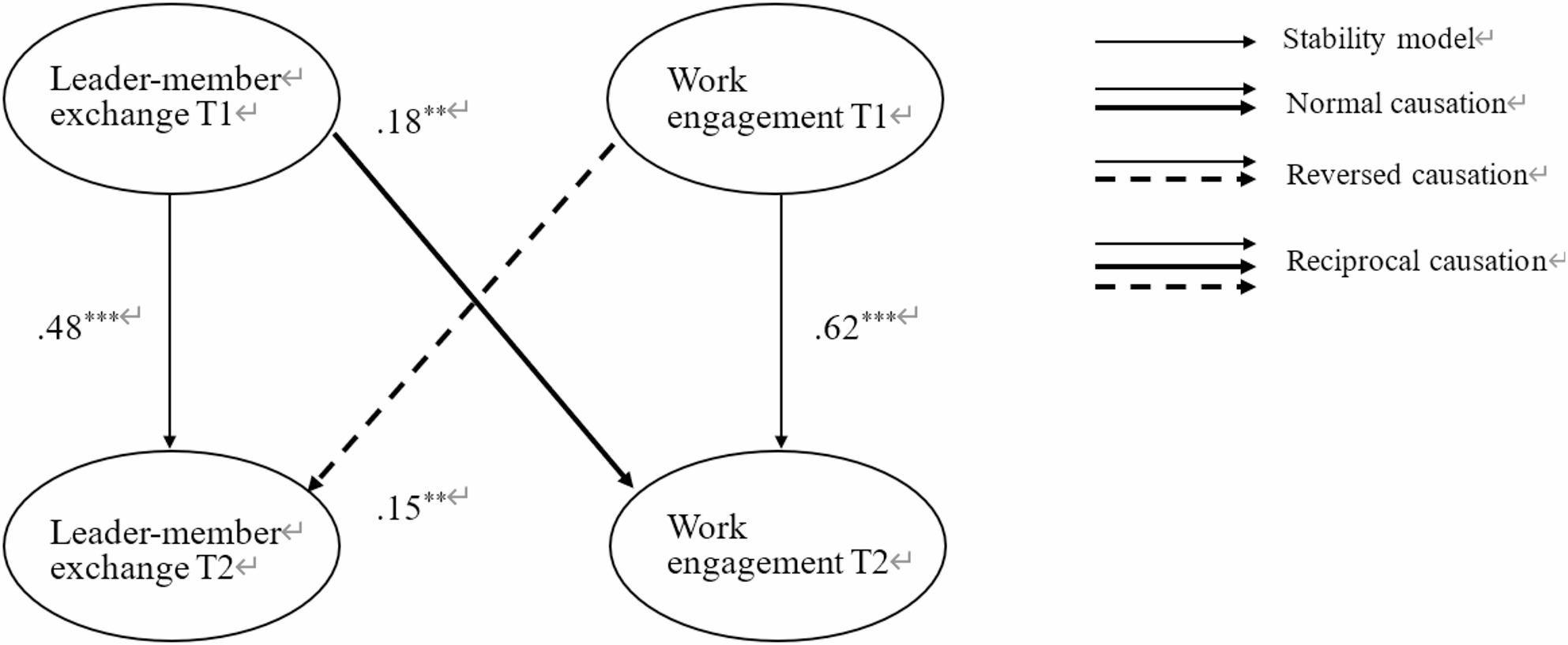
The final model of the statistically significant cross-lagged relationships between LMX and work engagement

Similarly, we then examined the cross-lagged relationships between Leader-Member Exchange (LMX) and work engagement. Table 4 demonstrates that the reciprocal model (M2reciproc), which posits bidirectional cross-lagged effects between LMX and work engagement, provided a better fit to the data than the stability model (M2stabil; ∆χ^2^ = 21.516, ∆*df* = 2, *p* < .001), the causality model (M2causal; ∆χ^2^ = 7.369, ∆*df* = 1, *p* < .01), and the reversed causation model (M2reversed; ∆χ^2^ = 9.633, ∆*df* = 1, *p* < .01). As depicted in Fig. 3, path analysis revealed that LMX at T1 significantly predicted work engagement at T2 (β = 0.18, SE = 0.06, *p* < .01), and conversely, work engagement at T1 significantly predicted LMX at T2 (β = 0.15, SE = 0.06, *p* < .01) after controlling the autoregression effect and the same demographical variables, indicating a reciprocal and positive relationship between LMX and work engagement over time.

In addition, we examined the direct relationships between Family Supportive Supervisor Behavior (FSSB) and work engagement. Model fit results showed that the reciprocal model representing cross-lagged effects between FSSB and work engagement has a better fit than the stability model (∆χ^2^ = 16.854, ∆*df* = 2, *p* < .001), and the reversed causation model (∆χ^2^ = 12.102, ∆*df* = 1, *p* < .001). However, the reciprocal model doesn’t show a significant better fit than the causal model (∆χ^2^ = 2.881, ∆*df* = 1, *ns*). In addition, a detailed path analysis found that FSSB at T1 positively predicts work engagement at T2 (β = 0.27, SE = 0.08, *p* < .001), whereas work engagement at T1 did not significantly predict future FSSB at T2 (β = 0.07, SE = 0.04, *ns*) in the reciprocal model after controlling relevant variables. Therefore, while FSSB appeared to positively influence work engagement, the reverse relationship was not true - work engagement did not significantly affect FSSB. Item loadings, composite reliability, and average variance extracted are shown in Table 5.

**Table 5:** Item loadings, composite reliability, and average variance extracted

Construct	Item	Std. Loading
Three-facor model at Time 1		
FSSB	Emotion support	0.760
	Instrumental support	0.671
	Role model	0.789
	Creative work-family management	0.812
	CR/AVE	0.845/0.577
Leader-member exchange	Item 1	0.575
	Item 2	0.765
	Item 3	0.344
	Item 4	0.676
	Item 5	0.770
	Item 6	0.718
	Item 7	0.772
	CR/AVE	0.849/0.457
Work engagement	Vigor	0.870
	Dedication	0.879
	Absorption	0.818
	CR/AVE	0.892/0.733
Three-facor model at Time 2		
FSSB	Emotion support	0.742
	Instrumental support	0.709
	Role model	0.835
	Creative work-family management	0.865
	CR/AVE	0.869/0.625
Leader-member exchange	Item 1	0.615
	Item 2	0.773
	Item 3	0.287
	Item 4	0.628
	Item 5	0.747
	Item 6	0.760
	Item 7	0.770
	CR/AVE	0.846/0.454
Work engagement	Vigor	0.867
	Dedication	0.901
	Absorption	0.843
	CR/AVE	0.904/0.758

Three-factor model means FSSB, Leader-member exchange, and work engagement

Finally, we tested the full cross-lagged model that includes FSSB, LMX, and work engagement. The comprehensive model shows a good model fit (*χ*^*2*^ = 748.981, *df =* 425, *p* < .001; IFI = 0.94, CFI = 0.94, TLI = 0.92). After controlling demographical variables and the autoregressive paths of each construct across time, the regression data found that FSSB at T1 positively predicts LMX at T2 (β = 0.31, SE = 0.15, *p* < .05), LMX at T1 positively predicts work engagement at T2 (β = 0.25, SE = 0.05, *p* < .001). Conversely, work engagement at T1 positively predicts LMX at T2 (β = 0.14, SE = 0.05, *p* < .01), LMX at T1 also positively predicts FSSB at T2 (β = 0.31, SE = 0.08, *p* < .001). Together, these findings strengthened the theoretical robustness of our proposed model and confirmed that FSSB, LMX, and work engagement are dynamically and mutually reinforcing over time. 

## Discussion

### Results conclusion

This two-wave cross-lagged panel study aimed to examine the gain spirals between supportive supervisor behavior (FSSB) and employees’ work engagement over time. Results confirmed our hypotheses. Gain spirals between supervisor and employee were found as FSSB predicted LMX and LMX predicted work engagement over time. In addition, work engagement positively influenced LMX, and LMX had a positive impact on future FSSB. These findings are consistent with several previous cross-sectional studies that have found positive associations between FSSB and LMX [10] and between LMX and work engagement [73, 74]. Theoretical and practical implications are discussed in the following sections.

### Theoretical implications

This research contributes to the managers’ supportive behavior literature from several perspectives. First, even though past research has highlighted that FSSB can facilitate work engagement [11], what is unique about the current study is that we found that a social relationship resource variable - here specified as LMX - served as a mediating mechanism [34]. This shows that FSSB can prompt employees’ work engagement through the improvement of interpersonal relationships. We used COR theory to explain the dynamic process of interpersonal resource flow, that is, work resources offered by supervisors could further prompt individuals’ resources engagement via enhancing relationship resources between supervisors and employees.

Second, to our knowledge, this research is the first longitudinal study that investigated a mediating mechanism between FSSB and work engagement. The novelty of this study was to focus on the positive feedback loops between supervisor and subordinate, thus expanding the notion of resource gain spirals. Existing research on gain spirals describes the relationships between intraindividual resource, and interindividual resources between coworkers [21, 67]. We found that the resources mutual beneficial relationship of organizational interpersonal level existed in not only peer-to-peer but also leader-to-member, which developed the research of resource gain spirals based on interpersonal level. Likewise, compared to former research targeting task-orientated job resources, we found that the supervisor behavior catering to employee family demands also had a mutual promotional effect on employee work engagement [7, 20]. FSSB prompted positive behaviors from employees through leader–member relationships, which further promoted the leader–member relationship and then encouraged supervisors to perform more FSSB. In addition, temporal reciprocal relationships between FSSB and LMX have already been reported in the earlier studies [63]. Our study showed that reciprocal effects between FSSB and LMX are also possible over longer periods of time. On the other hand, to our knowledge, the reciprocal effect between LMX and work engagement is a novel finding. LMX could positively predict work engagement, which was coherent with conclusions in former research [73, 74]. Work engagement would further improve the formation of high-level LMX, which conformed to the view of COR theory.

### Implications for practice

The findings from our study may be useful for practicing managers seeking to understand interactions between supervisors and their subordinates. The idea that FSSB targeted at employees can impact downstream LMX and work engagement, and is reciprocated in the form of work engagement back toward FSSB suggests that FSSB can permanently prompt employee work engagement. FSSB is a social support in the workspace and intervenable, which was demonstrated with intervention research that it could increase work satisfaction, decrease turnover intention, and enhance overall health [16, 33]. On one hand, organizations should integrate FSSB into their formal human resource practices to encourage managers and supervisors to enact supportive behaviors [10]. Specific to the supermarket condition, for example, we suggest that supermarkets can design and utilize performance evaluation indicators based on different dimensions of FSSB. In addition, given its critical feature of being labor-intensive, we encourage supermarkets to construct effective communication channels between employees and supervisors, thereby creating a supportive organizational climate beyond task-oriented management. On the other hand, FSSB has been demonstrated have a positive effect on employees’ well-being and family outcomes beyond the workplace [75]. Based on this insight, we further encourage supervisors to include more specified support regarding employees’ career self-development and family consideration (e.g., redesign work shifts to help employees take care of the old). Especially given that this study found that intervening in FSSB could cause a resource gain spiral that continuously motivated employee work engagement, we suggest supervisors actively assist employees to meet their diverse demands, release role stress, and result in more resources committed to work, improving work engagement and subjective well-being.

### Limitations and directions for future research

Some limitations of this study need to be acknowledged. First, we used self-reports to collect data, which may have led to bias because of common method variance. Despite our CFA results showing no strong common method bias, future studies would benefit from using various sources of data. For instance, given that the improvement of leader–member resource was a common perceptual result from both leader and employee [76], we suggest future research could invite both supervisors and members to evaluate the LMX scale [77]. In addition, we suggest that future research should collect data across a broader range of organizations and employ a multi-level analysis to further validate the robustness of the proposed gain spiral effect of FSSB on LMX and work engagement.

Another potential limitation of the present study is that we used repeated-measures data with two waves. According to Cole and Maxwell (2003), two-wave data can test the mediation effects when the relationship between variables meets the stationarity assumption over time [67]. However, we still encourage future research adopt a three-wave design to replicate our findings and give a more specific illustration of the mediation role of LMX. Moreover, future research could incorporate different moderators to further explore how these positive effects may change under specific conditions. Specifically, future research could investigate how leader flexibility and efficiency constraints in high-pressure environments shape the conditional provision of FSSB (e.g., the tightrope-walking leadership) [18]. Furthermore, future studies could also move beyond attitudinal outcomes to explore how these perceptual mechanisms drive potential differences that some employees reciprocate FSSB with great desire, while others may not, by using hybrid analytic approaches [42].

## Supplementary Information

Supplementary Material 1.

## Data Availability

The datasets presented in this study can be found in online repositories. The names of the repository/repositories and accession number(s) can be found in the article/supplementary material.
